# Adaptation to Hypoxia: A Chimera?

**DOI:** 10.3390/ijms21041527

**Published:** 2020-02-24

**Authors:** Michele Samaja, Giuseppina Milano

**Affiliations:** 1 Department of Health Science, University of Milano, I-20142 Milan, Italy; 2 Cardiovascular Research, Centre Hospitalier Universitaire Vaudois, 1011 Lausanne, Switzerland

**Keywords:** Adaptation, chronic hypoxia, intermittent hypoxia, hyperoxia, hypoxia mimetics, hypoxia antagonists, oxygen sensing, hypoxia-inducible factors, adaptation, high altitude, tumor microenvironment, pulmonary dysfunction, cardiovascular disease, apoptosis

“*The Chimera was, according to Greek mythology, a monstrous fire-breathing hybrid creature of Lycia in Asia Minor, composed of the parts of more than one animal [1]. Hence, the term "chimera" has come to describe anything composed of very disparate parts, or perceived as wildly imaginative, implausible, or dazzling*.”

[2]

A world-relevant clinical and environment issue that afflicts millions of people worldwide, hypoxia, i.e., the insufficient supply of oxygen (O_2_) with respect to demand, constitutes an important source of social and economic distress. However, despite the fact that hypoxia represents a potentially lethal condition, the human body possesses reserves that enable the recruitment of defense mechanisms to grant survival during relatively acute and/or amenable episodes. When hypoxia is chronic and/or severe, it is predicted to request a greater effort to balance its harmful effects, although the body may have a longer time to recruit gene-based and proteomic compensatory mechanisms. Chronic exposure to stressors, however, implies the concept of adaptation, or the “modification of an organism or its parts that makes it more fit for existence under the conditions of its environment: a heritable physical or behavioral trait that serves a specific function and improves an organism’s fitness or survival” (https://www.merriam-webster.com/dictionary/adaptation). Because at least some of the biological systems that constitute complex living matter have limited regenerative capacity, such as the cerebral and cardiopulmonary systems, the development of adaptation to hypoxia may show different features in different tissues. Studies on the molecular response to hypoxia, such as the overexpression of hypoxia-inducible factor-1 (HIF-1), were rewarded with the 2019 Nobel Prize in Physiology and Medicine. Ten contributors have recently examined various aspects of this fascinating issue in a Special Issue of The International Journal of Molecular Sciences.

The report by Sun et al. [3] focuses on autophagy, a cytoprotective mechanism well known to be triggered by hypoxia. This phenomenon was investigated in *M. nipponense*, a convenient animal model for this kind of study. According to previous literature, hypoxia upregulates the four mRNAs related to autophagy-related genes in a time-dependent manner in various tissues, but especially in the brain, liver and pancreas. As silencing of ATG4B (mainly expressed in the secretory and astrocyte cells of the brain) decreases cell viability in hypoxia, this report confirms in a novel model that autophagy is as an adaptive protective response to hypoxia.

The intestinal epithelium represents a paradigm of hypoxia adaptation because it is able to adapt to varying blood flows and, thus, large oxygen gradients due to its localization at the border between the anaerobic lumen and the mesenterial vessels. Still, adaptation may fail under pathologic situations that include hypoperfusion and inflammatory disease. The jejunum epithelium model was used by Dengler and Gable [4] to verify that the short-term adaptation to hypoxia is mediated by upregulation of the AMP-activated protein kinase (AMPK), which precedes the classical HIF-mediated adaptation, and acts primarily by modulating the transepithelial Na^+^-glucose cotransport via SGLT1. This observation adds to the complex interplay between hypoxia signaling and the various outcomes.

As for humans at altitude, the impact of metabolic syndrome seems specific to South American Indians, but not Tibetan populations. Indeed, Andean children display a prevalence of dyslipidemia and hypertension compared to dwellers at lower altitudes despite similar food intakes and daily activity levels, thereby indicating different metabolic adaptations. Barbacini et al. [5] investigated the sphingolipid pattern in underweight, normal weight, overweight and obese Andean children, showing that the high density lipoprotein–cholesterol axis may be related to hypoxia adaptation. Remarkably, the blood levels of ceramides and sphingosine-1-phosphate may represent a suitable circulating biomarker of increased risk of metabolic syndrome and cardiac dysfunction in young Andeans living at altitude.

Based on extensive cell culture and animal studies, the HIF-1 pathway, which is triggered by hypoxia, exercise and inflammatory stimuli, is often considered a hallmark of adaptation. Kammerer et al. [6] investigated the recruitment of the HIF-pathway in association with inflammation and exercise in non-acclimatized individuals exposed to acute hypobaric hypoxia. They found that both hypoxic and inflammatory stimuli are able to induce the HIF-1 signaling pathway in vitro, and that pro-inflammatory markers are associated with increased symptoms of acute mountain sickness, a paradigm of lack of adaptation. This contributes to the idea that HIF-1 overexpression may represent an index of maladaptation rather than adaptation.

Glioblastoma multiforme, the most aggressive and malignant primary brain tumor, often displays heterogeneous regions as a result of O_2_ gradients in the 0.1% to 10% range, which leads to increased aggressiveness and resistance. In a study performed with three glioblastoma cell lines cultured at 1% O_2_, Musah-Eroye and Watson [7] show that, compared to cells maintained in 20% O_2_, glioblastoma cells adapt to 1% O_2_ by reducing proliferation and enhancing metabolism, highlighting that hypoxia plays a pivotal role in changing the behavior of glioblastoma cells. Remarkably, this study also shows that genetic modulation can be reversed, supporting the concept of reversibility, and emphasizing that understanding the degree of O_2_ gradient in glioblastoma will be crucial in personalizing treatment.

Although physiological hypoxia (high altitude) may sometimes induce adaptation, pathological hypoxia (i.e., respiratory diseases) does not. Ottolenghi et al. [8] reviewed the hypoxia response in patients with high altitude pulmonary edema (HAPE), acute respiratory distress syndrome (ARDS), chronic obstructive pulmonary disease (COPD), and cystic fibrosis (CF), highlighting the hypoxia-dependent mechanisms that influence the prognosis of affected patients. It appears that iron metabolism (especially hepcidin) is highly involved in the compensatory response to hypoxia in healthy subjects; together with sphingolipid metabolism (especially ceramide and sphingosine-1-phosphate) they are active players of maladaptation to hypoxia.

Starting from the concept that hypoxia triggers ROS formation concomitantly with inflammation, and that hypoxia-mediated signaling upregulates the expression of the antioxidant transcription factor Nrf2, it is difficult to discern hypoxia from ROS stress. Paardekooper et al. [9] developed an optogenetic toolbox for organelle-specific ROS generation using a photosensitizer protein that produces superoxide anions upon excitation with 590 nm light, and can be fused to specific organelles to induce localized ROS bursts with high precision. It appears that selective ROS production does not affect cell viability in most organelles except for the nucleus. This may reveal a promising tool to induce locally targeted ROS production, opening up new possibilities to investigate processes and organelles that are affected by localized ROS production.

Intermittent hypoxic conditioning (IHC) is a promising approach to induce protection in several different tissues. Manukhina et al. [10] tested whether 14-day IHC, in addition to the known cardio-, vaso-, and neuroprotective effects, may also alleviate post-traumatic stress disorder, which causes mental and somatic diseases. IHC has favorable effects at several levels, including the anxiety index and the associated reduced glycogen content in the heart and liver, with reduced histological signs of metabolic and hypoxic damage and of impaired contractility. Liver and blood alanine and aspartate aminotransferase activities are also significantly decreased by IHC, together with the level of carbonylated proteins and lipid peroxidation products. Thus, IHC alleviated the injury caused by posttraumatic stress disorder, promoting IHC as a promising preventive treatment to reduce morphological and functional neurological damage.

A novel modification of the IHC paradigm, intermittent hypoxic–hyperoxic training (IHHT), consisting in four cycles of five minutes of hypoxia (12% FIO_2_) and three minutes of hyperoxia (33% FIO_2_), five times per week for three weeks (for a total of 15 sessions), improves cognitive test scores, along with a significant increase in amyloid precursor protein and decrease in amyloid beta expression and adhesion/activation of neutrophils in elderly patients with mild cognitive impairment. Serebrovska et al. [11] thus suggest a potential utility of IHHT as a new non-pharmacological therapy to improve cognitive function in pre-Alzheimer’s disease patients and slow down the development of Alzheimer’s disease.

Ottolenghi et al. [12] raise the question that, although commonly used in clinical practice to prevent or treat hypoxia, excess O_2_ (hyperoxia) may become toxic due to enhanced ROS formation that exceeds the antioxidant defenses and generates oxidative stress. They monitored such a response during and after general anesthesia in patients undergoing elective abdominal surgery who were kept at 40% and 80% O_2_ by measuring five oxidative stress biomarkers in blood samples. MDA, the main end product of the peroxidation of polyunsaturated fatty acids that is directly influenced by varying FIO_2_, may represent the best marker to assess the pro-oxidant/antioxidant equilibrium after surgery.

Although these competitive reports successfully address many aspects related to hypoxia adaptation, several unanswered or undeveloped questions still occur:

- How can we assess the onset of adaptation? In other words, which physiological (e.g., blunted erythropoiesis, control of alkalemia due to excess ventilation, recovery of homeostasis), molecular (e.g., normalization of NO stores, recruitment of hypoxia-sensitive genes and proteins), pathological (e.g., weight at birth, resistance against cardiopulmonary diseases, integrity of cerebral function) best represent a target of adaptation?
- Does adaptation invest the whole body, or can some organs/functions become better or faster adapted than others? This question may be linked to other important sub-questions: (1) Why are some populations better adapted than others, for example, Tibetan vs. South American dwellers? (2) How do Ethiopians, Kirgiz, and long-term inhabitants of Antarctica rank with respect to the mentioned Tibetan and South Americans dwellers? (3) Why do comparable degrees of hypoxia in some categories of patients lead to deleterious consequences, for example, blue babies and patients with COPD or pulmonary hypertension?
- Sometimes, high-altitude people opt to reside at lower altitudes. Can adaptation work in a reverse mode by enabling altitude-adapted people to survive relatively oxygen-rich atmospheres?
- Is adaptation always a positive factor, or are there instances where adaptation, or better maladaptation, might lead to deleterious patterns?
- Are factors such as the degree of activity, intermittent exposure to different oxygen levels, and life habits critical to enabling better and faster adaptation? Is there a role for hypoxia-induced oxidative stress in these patterns?
- Is the classical HIF-1 pathway sufficient to explain the complexity of the responses to chronic hypoxia and to enable adaptive patterns?
- Are appropriate biomarkers available to assess the degree of adaptation, or the lack of adaptation to hypoxia?

We believe that answering these questions may enable us to understand whether humans, who underwent genetic selection to be a low-altitude dwelling population, may ever be able to adapt to either environmental or pathological hypoxia, or if hypoxia adaptation is and will remain a chimera.
